# Update to the effectiveness and cost-effectiveness of a mindfulness training programme in schools compared with normal school provision (MYRIAD): study protocol for a randomised controlled trial

**DOI:** 10.1186/s13063-021-05213-9

**Published:** 2021-04-07

**Authors:** Jesus Montero-Marin, Elizabeth Nuthall, Sarah Byford, Catherine Crane, Tim Dalgleish, Tamsin Ford, Poushali Ganguli, Mark T. Greenberg, Obioha C. Ukoumunne, Russell M. Viner, J. Mark G. Williams, The MYRIAD team, Saz Ahmed, Matthew Allwood, Louise Auckland, Ruth Baer, Susan Ball, Marc Bennett, Sarah-Jayne Blakemore, Daniel Brett, Triona Casey, Katherine De Wilde, Darren Dunning, Eleanor-Rose Farley, Katie Fletcher, Lucy Foulkes, Cait Griffin, Kirsty Griffiths, Nils Kapplemann, Rachel Knight, Suzannah Laws, Jovita Leung, Liz Lord, Emma Medlicott, Lucy Palmer, Jenna Parker, Ariane Petit, Blanca Piera Pi-Sunyer, Isobel Pryor-Nitsch, Lucy Radley, Anam Raja, Ashok Sakhardande, Jeremy Shackleford, Anna Sonley, Laura Taylor, Maris Vainre, Lucy Warriner, Brian Wainman, Willem Kuyken

**Affiliations:** 1 Department of Psychiatry, University of Oxford, Warneford Hospital, Oxford, UK; 2 Kings Health Economics, Kings College London, London, UK; 3 Oxford Institute of Clinical Psychology Training, Warneford Hospital, Oxford, UK; 4 MRC Cognition and Brain Sciences Unit, University of Cambridge, Cambridge, UK; 5 Department of Psychiatry, University of Cambridge, Cambridge, UK; 6 Prevention Research Center, Pennsylvania State University, State College, PA USA; 7 NIHR ARC South West Peninsula (PenARC), University of Exeter Medical School, Exeter, UK; 8 UCL Institute of Child Health, London, UK

**Keywords:** Adolescence, Schools, Resilience, Mindfulness, Depression, Prevention, Protocol update

## Abstract

**Background:**

MYRIAD (My Resilience in Adolescence) is a superiority, parallel group, cluster randomised controlled trial designed to examine the effectiveness and cost-effectiveness of a mindfulness training (MT) programme, compared with normal social and emotional learning (SEL) school provision to enhance mental health, social-emotional-behavioural functioning and well-being in adolescence. The original trial protocol was published in Trials (accessible at 10.1186/s13063-017-1917-4). This included recruitment in two cohorts, enabling the learning from the smaller first cohort to be incorporated in the second cohort. Here we describe final amendments to the study protocol and discuss their underlying rationale.

**Methods:**

Four major changes were introduced into the study protocol: (1) there were changes in eligibility criteria, including a clearer operational definition to assess the degree of SEL implementation in schools, and also new criteria to avoid experimental contamination; (2) the number of schools and pupils that had to be recruited was increased based on what we learned in the first cohort; (3) some changes were made to the secondary outcome measures to improve their validity and ability to measure constructs of interest and to reduce the burden on school staff; and (4) the current Coronavirus Disease 2019 (SARS-CoV-2 or COVID-19) pandemic both influences and makes it difficult to interpret the 2-year follow-up primary endpoint results, so we changed our primary endpoint to 1-year follow-up.

**Discussion:**

These changes to the study protocol were approved by the Trial Management Group, Trial Steering Committee and Data and Ethics Monitoring Committees and improved the enrolment of participants and quality of measures. Furthermore, the change in the primary endpoint will give a more reliable answer to our primary question because it was collected prior to the COVID-19 pandemic in both cohort 1 and cohort 2. Nevertheless, the longer 2-year follow-up data will still be acquired, although this time-point will be now framed as a second major investigation to answer some new important questions presented by the combination of the pandemic and our study design.

**Trial registration:**

International Standard Randomised Controlled Trials ISRCTN86619085. Registered on 3 June 2016.

## Background

This update relates to the study protocol for ‘The effectiveness and cost-effectiveness of a mindfulness training programme in schools compared with normal school provision (MYRIAD): study protocol for a randomised controlled trial [1]’.

Mindfulness-based approaches for adults are effective at enhancing mental health [2], but few controlled trials have evaluated their effectiveness or cost-effectiveness for young people [3]. The primary aim of this trial is to evaluate the effectiveness and cost-effectiveness of a mindfulness training (MT) programme to enhance mental health, well-being and social-emotional-behavioural functioning in adolescence. To address this main aim, the design was a superiority parallel group cluster randomised controlled trial in which schools (clusters) offering social and emotional provision in line with good practice [4–6] were randomised to either continue this provision (control arm) or include MT in this provision (intervention arm). In general, this study will contribute to establishing if MT is an effective and cost-effective approach to promoting mental health in adolescence at school settings.

Through learning in the conduct of the trial set up and delivery, as well as the emergence of the coronavirus disease 2019 (SARS-CoV-2 or COVID-19) pandemic, the study team made several changes to the study protocol. This protocol update supplements the original study protocol1 and should be read in conjunction with it.

## Methods/design

Four major changes were made to the original study protocol [1]. All of them were discussed and agreed by the Trial Management Group and the Trial Steering Committee prior to their implementation, as well as receiving ethical approval from the University of Oxford Medical Sciences Inter-Divisional Research Ethics Committee.

### Elaboration of the school eligibility criteria

In the main study protocol, only schools that offer social and emotional learning (SEL) would be eligible for participation. It was specified for cohort 1 that schools were eligible for inclusion if their provision of SEL included, among others, a ‘written SEL policy’. However, for cohort 2, the ‘written SEL policy’ criterion was modified to ‘documentation denoting clear strategic planning of SEL within the school’. The original protocol stated that the initial eligibility criteria used in cohort 1 regarding SEL might be modified if necessary in subsequent cohorts. Experience in cohort 1 indicated that schools do not always use the term ‘SEL policy’ to denote strategic planning of SEL. Moreover, in some cases, there are schools that have an extensive, well-established and well-documented SEL curriculum, indicative of a clear structure and strategy around SEL, but do not have this formalised as a school policy. Therefore, we reworded this criterion as outlined above.

In the UK, many schools are often part of ‘academy chains’. To avoid contamination between the trial arms, if a school was part of an academy chain, normally, only one school per academy would be recruited, and schools already teaching mindfulness programmes were excluded from taking part in the trial for similar reasons.

### Changes in the number of schools and pupils recruited

It was stated in the first version of the protocol that the study would require 76 schools in total, with all year 7 and year 8 (or equivalent) students in each participating school (roughly 25,000 in total) being invited to take part in the baseline assessment. However, the project recruited extra schools to allow for dropout at this cluster level, predicting around 4 schools per study arm to drop put.

On the other hand, it was established that all participating schools randomised to MT would have to agree to deliver the programme to at least three different classes within years 8 and/or 9 (or equivalent), in order to have approximately 90 pupils in total per school to be eligible to participate in the full trial. This would provide 6840 pupils to be eligible to participate in the full trial, with the expectation that only 5700 would have provided parental consent or pupil assent, and around 4560 would complete the 2-year follow-up assessment. These assumptions were made based on data of two previous feasibility studies [7, 8], expecting that in every class of 30 pupils, 25 of them would have consent/assent to participate, and finally 20 of these would be followed up. These conditions would allow 75 participants per school proceeding to start full trial participation. However, as this sampling approach yielded a lower number of pupils in some schools in cohort 1, it was revised from three to four classes or more per school where possible in the second and larger cohort 2, with the aim to have approximately 100 pupils per school.

### Changes in secondary measures

During the course of the study, but before any specific data were collected, some changes were made to the secondary measures. The ‘Resistance to Peer Influence Scale’ (RPIS) that measures peer relationships [9] was removed after a pilot session completed with an Oxford-based young person’s advisory group. The questions were not easy for all pupils to understand, and it was felt the number of questions was too many for the pupils to complete in one school lesson.

When the project was first designed, the child-friendly EuroQol five dimensions measure (EQ-5D-Y) of health-related quality of life [10] was the best available measure for health economics studies with youth, but not totally suitable as it was adapted from an adult version of the questionnaire. It was finally replaced with the ‘Child Health Utility 9D’ (CHU-9D). The reason for making this change is that the CHU-9D is now considered a better scientific measure than the EQ-5-DY measure for use in young people. This is because the CHU-9D was developed using adolescents [11, 12], so it is a much better measure to use in this population. The CHU-9D has now been used in many studies and therefore there are value datasets available to use for the scoring of Quality of Life measures, allowing the calculation of quality-adjusted life years (QALYs) for use in economic evaluation.

In addition to the brief measure designed to assess risk behaviours, additional questions on binge drinking that were taken from the ESPAD study (http://www.espad.org) were added to broaden the items. The student level mindfulness practice and a pupil credibility assessment of SEL and MT lessons were also gathered, as well as two open questions about the possible existence of positive and negative experiences during or after the mindfulness lessons, as an opportunity to find out what the pupils felt about SEL and the course.

In cohort 2 at the 2-year follow-up (T4), we decided not to collect the teachers’ reports of the ‘Strengths and Difficulties Questionnaire’ (SDQ) [13] and the ‘Behaviour Rating Inventory of Executive Function’ (BRIEF-2) [14]. The reason was to try to reduce the burden on school staff who were under extraordinary pressure managing the impact of the COVID-19 pandemic. Moreover, this time-point was reframed in cohort 2 as a second major study to answer important new questions presented by the combination of the pandemic and our study design.

### Change of the primary endpoint to 1-year follow-up

The COVID-19 pandemic broke out in 2020, profoundly affecting every aspect of life in the UK from 30th March onwards. A nationwide lockdown started in March 2020, and all schools were instructed to close from 20 March 2020 although some schools remained open to the children of keyworkers and children deemed vulnerable by the senior leadership team. UK schools, teachers and children have all been affected in a range of ways, emotionally, socially and educationally. In relation to the MYRIAD trial, the pandemic broke out in the latter phase of the trial. For cohort 1 (12 schools), we had successfully completed all the data collection, so these data are pre-COVID-19 and unaffected by the pandemic. For the much larger cohort 2 (72 schools), the pandemic broke after the main MT intervention and after the T1, T2 and T3 data collection points, but before T4, the time point originally specified for collection of the primary outcome data.

Clearly, the most significant impact of the pandemic is therefore on the final primary outcome T4 data collection for cohort 2. Various scenarios have been considered, but all point to our cohort 2 T4 data collection point being seriously compromised by incomplete and poor quality data (e.g. the uncertainty about when/if schools will return and might return partially, researchers would not be able to go in the schools to administer the assessments due to social distancing issues, etc.), which would put in doubt the validity and precision of the estimates. Moreover, the findings have to be contextualised within the pandemic, and their generalizability beyond these particular circumstances is questionable. Therefore, there was an imperative to revise the protocol to ensure we can answer the research question we set out to answer originally.

Therefore, the primary endpoint will now be at 1-year (T3) rather than 2-year (T4) follow-up. The main scientific rationale for this is to enable us to ask the trial question using our stated design, without our findings being contingent on the pandemic and without our primary outcome data being seriously compromised. The T3 1-year follow-up was collected prior to the pandemic, in the same way across both trial arms. While this is a protocol change, it does not affect the basic trial question or design; it asks the main question at 1-year rather than 2-year follow-up. The original rationale for the 2 year follow-up being our primary endpoint was to extend as much as possible the time point to measure long term effects of this universal MT programme [15]. In this sense, changing the primary time point to T3 reduces the possibility of evaluating long term effects, which is something relevant in the current state of the field [3, 16] but with the counterpart of ensuring a reliable answer to our primary question. Nevertheless, the longer T4 follow-up data will still be acquired for cohort 2, but this time-point will be secondary outcome, interpretable only within the context of the issues above. The sample size calculation did not state the timing of the end point, so these calculations are equally valid for the T3 follow-up as the T4 follow-up.

## Conclusion

In order to improve the clarity of school eligibility criteria, one criterion related to the description of provision of SEL at schools was developed with more detail. In addition, according to the conduct observed in cohort 1, the necessary number of schools and pupils recruited was extended to ensure an adequate sample size. Also, to recognise the development of new and better measures and our own learning in cohort 1, some of the instruments were changed and new measures were included. Moreover, to reduce the burden on school staff, two teacher rated scales of pupils were removed. Finally, because the pandemic put at risk the ability of the study to answer the proposed questions using the original primary endpoint, it was changed from 2-year follow-up to 1-year follow-up. This change reduced the possibility of assessing long term effects associated with the programme. However, the 1-year follow-up assessment eliminates the risk of data loss and confounding because it is a pre-pandemic measurement.

### Trial status

Recruitment of schools was completed in Autumn 2017. The first cohort of schools (12) completed their 2-year follow-up visits in January 2020. The second cohort of schools (72) completed the 1-year follow-up of the project during the Autumn term of 2019 with some schools finishing in January 2020. The final 2-year follow-up was planned to take place in Autumn 2020; however, due to the outbreak of COVID-19, at the time of writing the timing of this phase of data collection remain uncertain and will be moved to a later time point.

## Data Availability

Data and materials (codebook, statistical analysis plan, protocols, etc.) are available from Prof. Kuyken (willem.kuyken@psych.ox.ac.uk) upon request (release of data will be subject to an approved proposal and a signed data access agreement).

## Abbreviations

BRIEF-2: Behaviour Rating Inventory of Executive Function version 2
CHU-9D: Child Health Utility 9D
EQ-5D-Y: Child-friendly EuroQol five dimensions measure of health-related quality of life
ESPAD: European School Survey Project on Alcohol and Other Drugs
MYRIAD: My Resilience in Adolescence
MT: Mindfulness training
QALYs: Quality-adjusted life years
RPIS: Resistance to Peer Influence Scale
SEL: Social and emotional learning
SARS-CoV-2 or COVID-19: Coronavirus disease 2019
SDQ: Strengths and Difficulties Questionnaire
Year 7 (England and Wales): Pupils aged 11–12 years
Year 8 (England and Wales): Pupils aged 12–13 years
Year 9 (England and Wales): Pupils aged 13–14 years
